# Marine Predators Algorithm for Forecasting Confirmed Cases of COVID-19 in Italy, USA, Iran and Korea

**DOI:** 10.3390/ijerph17103520

**Published:** 2020-05-18

**Authors:** Mohammed A. A. Al-qaness, Ahmed A. Ewees, Hong Fan, Laith Abualigah, Mohamed Abd Elaziz

**Affiliations:** 1 State Key Laboratory for Information Engineering in Surveying, Mapping and Remote Sensing, Wuhan University, Wuhan 430079, China; alqaness@whu.edu.cn; 2 Department of e-Systems, University of Bisha, Bisha 61922, Saudi Arabia; ewees@du.edu.eg; 3 Department of Computer, Damietta University, Damietta 34517, Egypt; 4 Faculty of Computer Sciences and Informatics, Amman Arab University, Amman 11953, Jordan; aligah.2020@gmail.com; 5 Department of Mathematics, Faculty of Science, Zagazig University, Zagazig 44519, Egypt; abd_el_aziz_m@yahoo.com

**Keywords:** COVID-19, ANFIS, SARS-CoV-2, forecasting, marine predators algorithm (MPA)

## Abstract

The current pandemic of the new coronavirus, severe acute respiratory syndrome coronavirus 2 (SARS-CoV-2), or COVID-19, has received wide attention by scholars and researchers. The vast increase in infected people is a significant challenge for each country and the international community in general. The prediction and forecasting of the number of infected people (so-called confirmed cases) is a critical issue that helps in understanding the fast spread of COVID-19. Therefore, in this article, we present an improved version of the ANFIS (adaptive neuro-fuzzy inference system) model to forecast the number of infected people in four countries, Italy, Iran, Korea, and the USA. The improved version of ANFIS is based on a new nature-inspired optimizer, called the marine predators algorithm (MPA). The MPA is utilized to optimize the ANFIS parameters, enhancing its forecasting performance. Official datasets of the four countries are used to evaluate the proposed MPA-ANFIS. Moreover, we compare MPA-ANFIS to several previous methods to evaluate its forecasting performance. Overall, the outcomes show that MPA-ANFIS outperforms all compared methods in almost all performance measures, such as Root Mean Squared Error (RMSE), Mean Absolute Error (MAE), Mean Absolute Percentage Error (MAPE), Root Mean Squared Relative Error (RMSRE), and Coefficient of Determination($R^{2}$). For instance, according to the results of the testing set, the $R^{2}$ of the proposed model is 96.48%, 98.59%, 98.74%, and 95.95% for Korea, Italy, Iran, and the USA, respectively. More so, the MAE is 60.31, 3951.94, 217.27, and 12,979, for Korea, Italy, Iran, and the USA, respectively.

## 1. Introduction

Coronaviruses are a family of viruses that are serious pathogens of people. They result in gastrointestinal, hepatic, neurological, and severe respiratory diseases. Their main distributions are among humans, bats, mice, livestock, and wild animals [1,2,3]. The last two decades witnessed three outbreaks of coronaviruses, called SARS-CoV, MERS-CoV, and SARS-CoV-2 (COVID-19), in 2003, 2012, and 2019, respectively. These three outbreaks have confirmed human-to-human and animal-to-animal transmission [4].

According to the official numbers of the confirmed cases of the three mentioned outbreaks, the new coronavirus, COVID-19, is the most dangerous, and its spread is the highest, as recorded in more than 200 countries and territories. The first reported cases of COVID-19 were recorded in Wuhan City, Hubei Province, China [5]. The beginning was linked to several people who visited a local seafood market in Wuhan and suffered from respiratory illness. The number of reported cases increased daily in Wuhan, Hubei province, and in other Chinese cities and provinces. After a short time, several countries recorded confirmed cases of COVID-19, such as Japan, Korea, and several other countries. Thereafter, a huge outbreak of COVID-19 spread in many countries, especially in European countries, such as Italy, Spain, Germany, France, and others. In Asia, except China, the most affected countries are Korea and Iran; whereas in the Americas, the most affected country is the USA. The source of the new coronavirus, COVID-19, is still unconfirmed, and in some studies, such as Lu et al. [6], it was shown that bat-derived coronavirus strains were similar to COVID-19; therefore, the authors found that bats were the potential source of COVID-19.

The daily confirmed cases globally have sharply increased, even with the strict policies implemented by governments and the lockdown of many cities in the world. The main reason for that is the incubation period of COVID-19, which may be up to 14 days, as described by Chen et al. [7]. During the incubation period, the infection can be transmitted to others even if the infected person does not have symptoms. Furthermore, for some people, the incubation period may reach 24 days, as concluded by Guan et al. [8].

The rapid spread of COVI-19 confirms that it is a terrifying pandemic; therefore, it is necessary to study and analyze the increase of the affected cases or so-called confirmed cases.

Forecasting previous epidemics has received wide attention, and different methods have been proposed. For example, a forecasting model based on Bayesian inference was proposed by Shaman et al. [9] to forecast the outbreaks of Ebola in Guinea, Liberia, and Sierra Leone. An ensemble adjustment Kalman filter based forecasting method was proposed by Shaman et al. [10] to forecast seasonal outbreaks of influenza in New York City. Another Kalman filter based model was also proposed by Shaman et al. [11] to forecast weekly influenza cases. Moreover, different mathematical and statistical methods have been proposed for various epidemics, such as hepatitis A virus infection proposed by True and Kurt [12], West Nile virus (WNV), proposed by Defelice et al. [13], SARS proposed by Massad et al. [14], influenza A (H1N1-2009) proposed by Ong et al. [15], and MERS proposed by Nah et al. [16].

Recently, there have been several studies presented to address different forecasting issues for COVID-19, for example: forecasting of the human-to-human transmission of COVID-19 by Thompson [17], forecasting the number of confirmed cases of COVID-19 by Zhao et al. [18] and Al-qaness et al. [19], forecasting the infection rate of COVID-19 by Nishiura et al. [20], estimating the transmission risk of COVID-19 by Tang et al. [21], and estimating the risk of death of COVID-19 by Jung et al. [22].

On 24 March 2020, the number of confirmed COVID-19 cases reached 24,811, 69,176, 9073, and 53,740 in Iran, Italy, Korea, and the USA, respectively. In this paper, we propose a time-series forecasting approach to forecast confirmed cases of COVID-19 in four countries, Korea, the USA, Italy, and Iran, using an improved adaptive neuro-fuzzy inference system (ANFIS). The ANFIS is a well-known time-series forecasting model, which has received wide attention and been applied for various prediction and forecasting issues, such as stock prices [23], oil prices [24], energy and oil consumption [25,26,27], and others. One of the main limitations of ANFIS is the estimation of its parameters. Recently, various optimization approaches were employed to solve this challenge, such as the sine-cosine algorithm (SCA) [26], particle swarm intelligence (PSO) [28,29,30], and social-spider optimization [31].

In this paper, we present an improved ANFIS version, by enhancing its performance using a new nature-inspired optimization approach, called the marine predators algorithm (MPA). The MPA was proposed by Faramarzi et al. [32]. It is inspired by the foraging strategy of ocean predators, based on two types of strategies, called Lévy and Brownian motion, which are selected by the predators for optimal foraging. Therefore, in this study, we leverage the MPA to optimize the ANFIS parameters.

In our previous study [19], we proposed an enhanced ANFIS forecasting model, called FPASSA-ANFIS. We forecasted the number of infected people in China. Although the proposed model showed good performances, using two metaheuristics, salp swarm algorithm (SSA) and flower pollination algorithm (FPA), was a little complex. However, it was found that it needs more improvements, especially to deal with large-scale datasets, and also, its exploration ability is less effective than its exploitation. Therefore, this study applied a new metaheuristic method called the marine predators algorithm (MPA) [32]. This algorithm simulates the strategy that represents the relation between the predator and prey in the ocean by using the Brownian and Lévy movements. Our developed MPA-ANFIS approach begins by setting the initial value for its parameters. Then, this is followed by splitting the historical data of COVID-19 for the specified country into two sets of training and testing. Then, we set the initial value for a set of solutions that indicate the configuration of the parameters of the ANFIS network. Thereafter, we compute the performance of the ANFIS model using the training set and the current configuration/solution using the root mean squared error (RMSE) as an objective function. The next step is to determine the best configuration of the parameter. We then use the operators of MPA to update the other solutions. After reaching the terminal condition, the best solution is used to build the ANFIS model and the testing set to assess the constructed ANFIS model. This next step is the forecasting of COVID-19.

The primary contributions and objectives are listed as follows:

1. We propose a robust time-series model for forecasting the number of infected people (confirmed cases) of SARS-CoV2 in several countries, Iran, Italy, Korea, and the USA.
2. We improve the performance of the ANFIS model using a novel optimization method, MPA, which has not been applied in previous studies since the MPA is a new algorithm proposed in recent months.
3. We evaluate the proposed MPA-ANFIS with official datasets and by comparing it with several previous forecasting methods.

The rest of sections of this study are arranged as follows: Section 2 consists of the preliminaries of ANFIS and MPA. Section 3 presents the MPA-ANFIS method. Experiments and results are described in Section 4. Finally, the conclusion is presented in Section 6.

## 2. Preliminaries

### 2.1. Adaptive Neuro-Fuzzy Inference System

**Figure 1:**
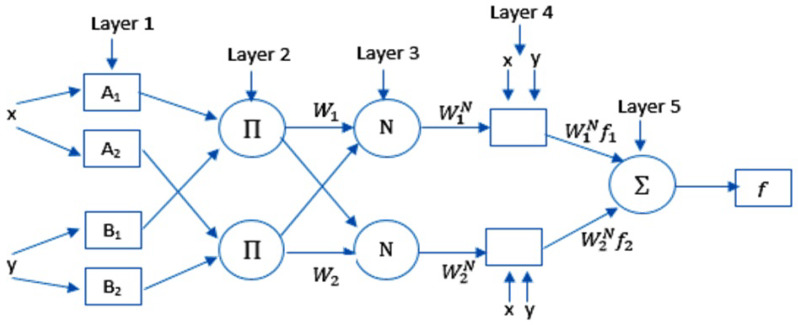
The basic structure of the ANFIS model.

In general, ANFIS creates a mapping between inputs and outputs by employing “IF-THEN rules” (also known as the “Takagi–Sugeno inference model”). The basic structure of ANFIS is shown in Figure 1. As shown in this figure, the inputs of Layer 1 are represented by *x* and *y*, where the output of node *i* is represented by $O_{1i}$, as follows:

(1) $$O_{1i}=\mu_{A_{i}}\left(x\right),\;i=1,2,\;O_{1i}=\mu_{B_{i-2}}\left(y\right),\;i=3,4$$

(2) $$\mu \left(x\right)=e^{-{\left(\frac{x-\rho_{i}}{\alpha_{i}}\right)}^{2}};$$

hence, μ is the generalized Gaussian membership function. The membership values of μ are represented by $A_{i}$ and $B_{i}$. The premise parameter set is represented by $\alpha_{i}$ and $\rho_{i}$.

Moreover, Equation (3) defines the output of Layer 2:

(3) $$O_{2i}=\mu_{A_{i}}\left(x\right)\times \mu_{B_{i-2}}\left(y\right)$$

Equation (4) defines the output of Layer 3:

(4) $$O_{3i}={\bar{w}}_{i}=\frac{\omega_{i}}{\sum_{\left(i=1\right)}^{2}\omega_{i}},$$

where $w_{i}$ is the *i*th nodes output from the previous layer.

The output of Layer 4 is represented by Equation (5):

(5) $$O_{4,i}={\bar{w}}_{i}f_{i}={\bar{w}}_{i}\left(p_{i}x+q_{i}y+r_{i}\right)$$

where where *f* is a function that combines the inputs and parameters of network. The consequent parameters of node *i* are represented by $r_{i}$, $q_{i}$, and $p_{i}$.

Finally, the output of Layer 5 is represented by Equation (6):

(6) $$O_{5}=\sum_{i}{\bar{w}}_{i}f_{i}$$

### 2.2. Marine Predators Algorithm

In this section, the formulation of the marine predators algorithm is introduced [32]. Similar to other metaheuristic (MH) techniques, the MPA starts by assigning random values for a set of solutions depending on the search space, and this is formulated as:

(7) $$X=LB+r_{1}\times \left(UB-LB\right)$$

In Equation (7), LB refers to the lower boundary in the search space, while UB is the upper boundary. $r_{1}\in \left[0,1\right]$ is the random number. The MPA has a strategy that considers the prey and predator as a search agent since when the predator searches for the prey, the prey itself is searching for its food. Therefore, the elite (matrix of the top predators) will be updated at the end of each generation. The formulation of the elite and prey (*X*) is given as [32]:

(8) $$Elite=\left[\begin{array}{cccc} X_{11}^{1} & X_{12}^{1} & \ldots & X_{1d}^{1}\\ X_{21}^{1} & X_{22}^{1} & \ldots & X_{2d}^{1}\\ \ldots & \ldots & \ldots & \ldots \\ X_{n1}^{1} & X_{n2}^{1} & \ldots & X_{nd}^{1} \end{array}\right],\;X=\left[\begin{array}{cccc} X_{11} & X_{12} & \ldots & X_{1d}\\ X_{21} & X_{22} & \ldots & X_{2d}\\ \ldots & \ldots & \ldots & \ldots \\ X_{n1} & X_{n2} & \ldots & X_{nd} \end{array}\right],\;$$

The next step is to update the position of prey *X*, which is performed using three stages depending on the variant ratio of velocity simultaneously emulating the entire relation between prey and predator. The details of each stage are discussed in the following subsections.

#### 2.2.1. Stage 1: High-Velocity Ratio

In this stage, the predator is moving faster than *X* in the exploration phase, and this occurs in the first third of the total number of generations (i.e., $\frac{1}{3}t_{max}$). Therefore, the prey $S_{i}$ is updated using the following equations.

(9) $$S_{i}=R_{B}⊗\left(Elite_{i}-R_{B}⊗X_{i}\right),i=1,2,\ldots ,n$$

(10) $$X_{i}=X_{i}+P.R⊗S_{i}$$

where R∈[0,1] and P=0.5 represent a vector of uniform random numbers and a constant number, respectively. $R_{B}$ represents a random vector that refers to the Brownian motion. ⊗ indicates the process of element-wise multiplications.

#### 2.2.2. Stage 2: Unit Velocity Ratio

In this stage, the prey and predator are moving in the same area, and this movement simulates the process of searching for the prey/food. Furthermore, this refers to the process of changing the status of the MPA from exploration to exploitation. Actually, both of them have the same chance to occur during this stage. Following [32], exploration is performed using the predator, while exploitation is performed by the prey. It is assumed that the Lévy flight and Brownian motion represent the prey movement and the predator, respectively, and this is defined as in Equations (11) and (12) when $\frac{1}{3}t_{max}<t<\frac{2}{3}t_{max}$:

(11) $$\begin{array}{c} S_{i}=R_{L}⊗\left(Elite_{i}-R_{L}⊗X_{i}\right),i=1,2,\ldots ,n \end{array}$$

(12) $$\begin{array}{c} X_{i}=X_{i}+P.R⊗S_{i} \end{array}$$

where $R_{L}$ represents random numbers following a Lévy distribution. Equations (11) and (12) are applied to the first half of the population that represents the exploitation. While for the second half of the population:

(13) $$S_{i}=R_{B}⊗\left(R_{B}⊗Elite_{i}-X_{i}\right),i=1,2,\ldots ,n$$

(14) $$X_{i}=X_{i}+P.CF⊗S_{i},\;CF={\left(1-\frac{t}{t_{max}}\right)}^{2\frac{t}{t_{max}})}$$

where CF is the parameter that controls the step size of movement for the predator and $t_{max}$ represents the total number of generations.

#### 2.2.3. Stage 3: Low-Velocity Ratio

This stage is the last process in the optimization process, which occurs when the movement of the predator is faster than the prey. This refers to the exploitation phase when $t>\frac{2}{3}t_{max}$, and this is formulated as:

(15) $$S_{i}=R_{L}⊗\left(R_{L}⊗Elite_{i}-X_{i}\right),i=1,2,\ldots ,n$$

(16) $$X_{i}=X_{i}+P.CF⊗S_{i},\;CF={\left(1-\frac{t}{t_{max}}\right)}^{2\frac{t}{t_{max}})}$$

#### 2.2.4. Eddy Formation and FADs’ Effect

There are issues of the environment that affect the behavior of marine predators such as fish aggregating devices (FADs). The effect of FAD is formulated as:

(17) $$X_{i}=\left\{\begin{array}{cc} X_{i}+CF[X_{min}+R⊗\left(X_{max}-X_{min}\right)⊗U & r_{5}<FAD\\ X_{i}+\left[FAD\left(1-r\right)+r\right]\left(X_{r1}-X_{r2}\right) & r_{5}>FAD \end{array}\right.$$

In Equation (17), FAD=0.2, and *U* is a binary solution, and this is preformed by generating a random solution, then converting it to a binary solution using the threshold 0.2. r∈[0,1] represents a random number. $r_{1}$ and $r_{2}$ are the indices of the prey.

#### 2.2.5. Marine Memory

Following [32], the marine predator has a memory that remembers the good position that it has reached. In general, the fitness value of each solution is compared with the previous fitness value, and the best one is saved in memory. The pseudo-code of MPA is presented below.

## 3. The Proposed Method

This section introduces the proposed method called PMA-ANFIS. The goal of PMA-ANFIS is to forecast the number of cases of COVID-19 in four countries, namely Italy, the USA, Iran, and Korea.

The proposed method improves ANFIS by optimizing its parameters. The ANFIS model was selected because it is widely used in many forecasting tasks. It also can work effectively with uncertainty, fuzziness, and ambiguity in the problem. MPA is a new optimization algorithm; it shows good performance in selecting the best ANFIS parameters compared to other methods.

PMA-ANFIS is constructed using the five layers of the ANFIS model, where the Layer 1 receives the input data, and Layer 5 produces the results. The main goal of FPA is to optimize the ANFIS weights that lie between Layers 4 and 5. This process works in the training phase.

PMA-ANFIS receives the number of confirmed cases and their dates. Then, the input data are formed by the proposed method to be in a time-series format. Due to the data diversity in the four countries, the autocorrelation function (ACF) is applied to perform this step. It searches for patterns in the data and helps select the best one. It is recommended that a number greater than 0.2 be considered; therefore, in this study, 6 lags were selected for the USA dataset, 5 lags for both the Korean and Iranian datasets, and 7 lags for the Italian dataset. With these settings, the input data were formed.

The entire dataset was divided into two groups. The first group (i.e., training set) contained 75% of the data, while the rest was used as a testing set. ANFIS applies the fuzzy c-means method, and the cluster number was set to seven.

To evaluate the quality of the candidate parameters, the mean squared error (MSE) was applied (as in Equation (18)). The MSE computes the error between the target and the produced data.

(18) $$MSE=\frac{1}{N_{a}}\sum_{i=1}^{N_{s}}{\left(g_{i}-d_{i}\right)}^{2}$$

where *g* indicates the target data. *d* is the output of the produced data. The size of the population is defined by the variable $N_{a}$.

As the optimization method, MPA-ANFIS starts by creating a population (*X*) to represent the problem population. After that, the objective function is applied to test the solutions individually. In each iteration, the value of the MSE is checked, and the solution that has the lowest value of MSE is saved as the best solution. MPA-ANFIS works and loops its steps until meeting the stop criterion, and the best parameter of ANFIS is passed to the testing stage. The optimized ANFIS model is used to compute the final results in the testing stage.

**Figure 2:**
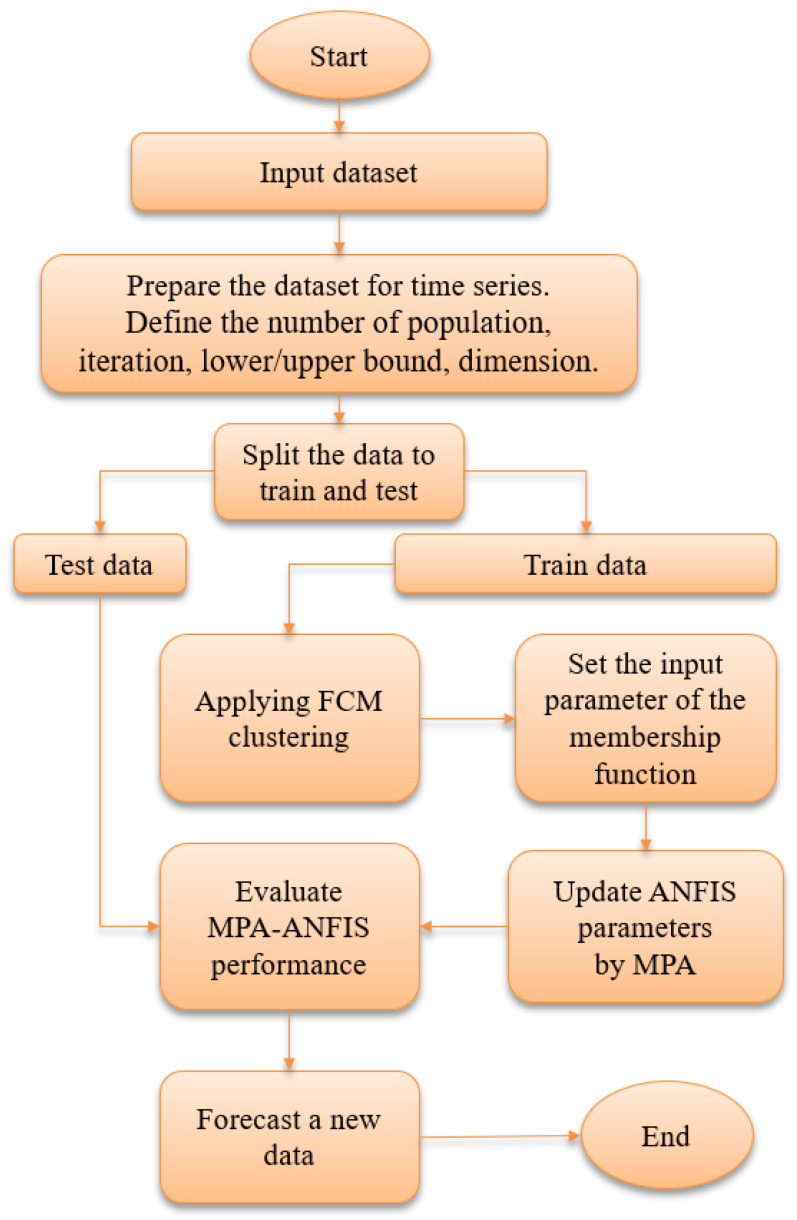
The flowchart of the proposed MPA-ANFIS algorithm. FCM, the Fuzzy c-means.

MPA-ANFIS was evaluated using well-known performance measures, namely root mean squared error (RMSE), mean absolute error (MAE), mean absolute percentage error (MAPE), and coefficient of determination ($R^{2}$). The MPA-ANFIS stages are illustrated in Figure 2.

## 4. Experiment and Results

### 4.1. Data

We used the datasets of reported cases of COVID-19 in four countries. They were obtained from the website of the World Health Organization (WHO) [5]. The datasets included the daily confirmed cases in four countries, the USA, Korea, Iran, and Italy. The total days for each country equaled 77 days, from 22 January 2020 to 7 April 2020. Seventy-five percent of the dataset was applied to train the proposed method, and the rest was applied in the testing phase.

### 4.2. Performance Measures and Parameter Setting

In this study, a set of metrics was used to assess the MPA-ANFIS approach and other models. These metrics are defined in Table 1.

In Table 1, $N_{s}$, Yp, and *Y* are the number of samples, the original COVID-19 dataset, and its prediction, respectively. The average of *Y* is given by $\bar{Y}$. The model that had the smallest values for the metrics (except a high value for $R^{2}$) was the best one.

In addition, Table 2 shows the value for all compared algorithm, including original adaptive neuro-fuzzy inference system (ANFIS), and enhanced ANFIS with genetic algorithm (GA), particle swarm optimizer(PSO), Artificial bee colony (ABC), the hybridized of flower pollination algorithm and salp swarm algorithm (SSAFPA), sine-cosine algorithm (SCA) that were used in our comparison. There were general parameters that would be used over all the tested algorithms, such as the number of solutions was set to 25. The total number of generations was 100; also, each algorithm was performed 30 times in independent runs [26,27,33,34]

### 4.3. Results

The comparison results between the forecasting COVID-19 model based on MPA-ANFIS and other models are given in Table 3, Table 4, Table 5 and Table 6 based on the testing set (where the bold results showed the best results). By analyzing the USA dataset, it can be observed that MPA predicted the number of cases confirmed for COVID-19 nearly the same as the target number since it had the smallest RMSE, MAE, MAPE, and RMSRE, as well as it had the highest $R^{2}$. The performance of other models was different according to the performance measures.

By analyzing the performance of the MPA-ANFIS model using the Iranian dataset, it could be noticed that it provided better results than others among all measures except the RMSE, which was allocated to the second rank after GA. In addition, it can be seen that PSO, GA, and MPA nearly had the same performances, but MPA was allocated the first rank. Furthermore, the other three models (i.e., ABC, SCA, and FPASSA) nearly had the same behavior for Iran, except SCA was the lowest in terms of $R^{2}$, which provided nearly 20%.

In the case of the performance of the proposed model to predict COVID-19 for Italy, it could be observed that MPA had better results in terms of RMSE, MAE, MAPE, and $R^{2}$. However, in terms of RMSRE, GA provided the smallest value, followed by PSO and MPA, which were the second and third rank, respectively.

Finally, when Korea’s COVID-19 dataset was used, it could be noticed that in terms of MAE, MAPE, and $R^{2}$, GA based on ANFIS was the best algorithm. However, in other terms (i.e., RMSE and RMSRE), the developed MPA-ANFIS was better than the others.

**Figure 3:**
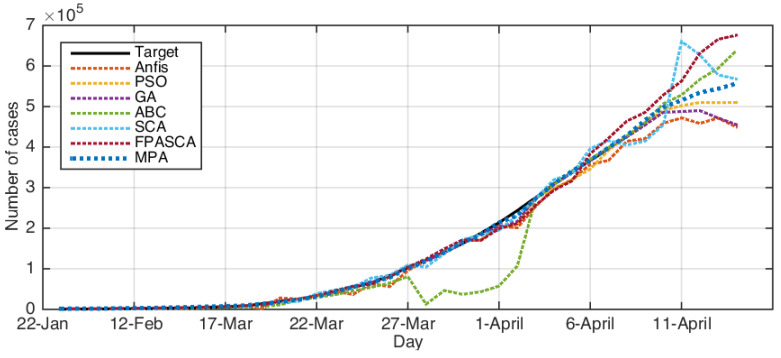
The results for the USA using ABC, ANFIS, FPASSA, GA, MPA, SCA, and PSO against real data (target).

**Figure 4:**
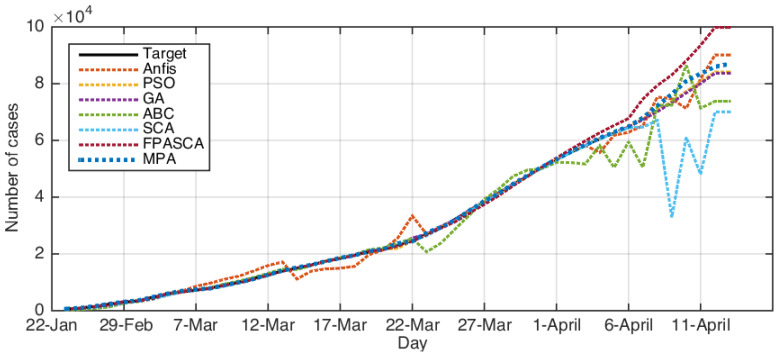
The results for Iran using ABC, ANFIS, FPASSA, GA, MPA, SCA, and PSO against real data (target).

**Figure 5:**
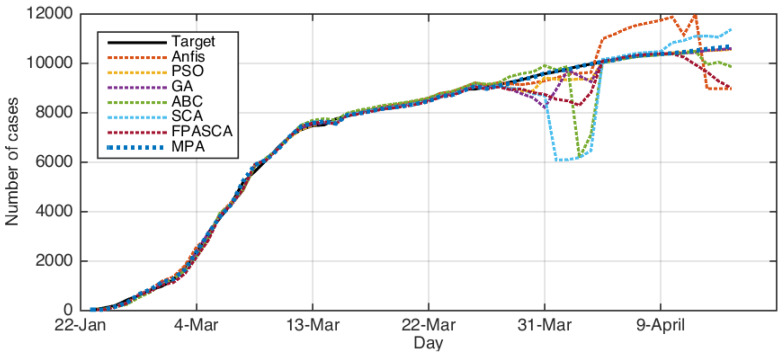
The results for Korea using ABC, ANFIS, FPASSA, GA, MPA, SCA, and PSO against real data (target).

**Figure 6:**
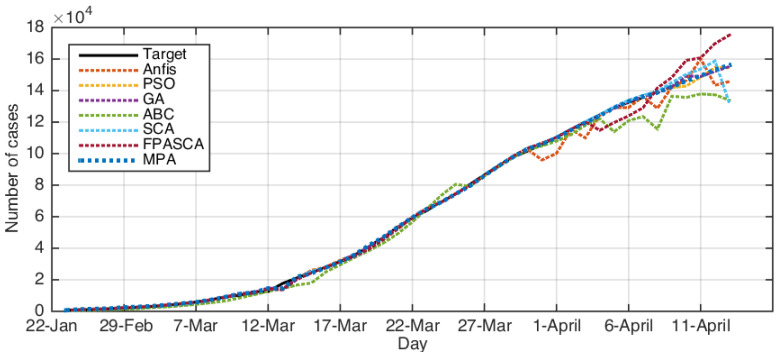
The results for Italy using ABC, ANFIS, FPASSA, GA, MPA, SCA, and PSO against real data (target).

Figure 3, Figure 4, Figure 5 and Figure 6 depict the original COVID-19 dataset and the forecasting for each country. It can be seen from Figure 3 that the overall common forecasting by the prediction methods for COVID-19 for the USA would be growth; therefore, the USA government needs to implement stricter policies to reduce the infection. For Iran (as in Figure 4), the forecasting of COVID-19 among all methods indicated that the situation would still be growth, except SCA, which predicted that it would go down and become nearly stable after several days; however, we ignored the SCA results because its $R^{2}$ was not good and the RMSE was very high. Therefore, our recommendation for Iran was to put more restrictions on people’s movement and maintain social distancing since this is one of the greatest problems facing Middle-Eastern countries. From Figure 6, which represents the forecasting of COVID-19 for Italy, it could be noticed from the best algorithm MPA, PSO, GA, and FPSSA that COVID-19 will have an exponential growth. For Korea, as in Figure 5, it could be observed that the situation already became stable.

## 5. Discussion

In this paper, we proposed a modified ANFIS model using a new optimization algorithm, called MPA, to forecast the number of confirmed cases of COVID-19 in four different countries, Italy, Iran, Korea, and the USA.

By analyzing the relation of confirmed cases (RCC) between the confirmed cases and the four countries’ areas, we could note that there was a positive relation in all countries. The area of Italy was the smallest one among the four countries (301,339 $km^{2}$), and the RCC was the highest one, equaling 10.29%, whereas, the USA had the largest area (952,5067 $km^{2}$), and the RCC was the smallest one, equaling 0.44%. The RCC of Korea (100,210 $km^{2}$) was 4.25%, and the RCC of Iran (164,8195 $km^{2}$) was 1.13%.

From the analysis of forecasting confirmed COVID-19 cases for the four countries, it could be observed that the confirmed cases rate increased between 2% and 42% in Italy and between 8% and 40% in the USA, whereas, in Iran and Korea, it increased between 3% and 13% and 0.5% and 3%, respectively.

In this study, we proposed an alternative forecasting COVID-19 model that depended on improving the quality of the ANFIS model using MPA. The proposed MPA used the COVID-19 datasets from four countries. The main aim of using those datasets was to test the ability of ANFIS-MPA to work with data collected from different countries, and each one of these countries had its dynamics and different internal conditions.

The results of the improved ANFIS using MPA seemed to propose that the COVID-19 curve for the USA, Iran, and Italy had an exponential form, and for Korea after 13 March, it increased with small numbers. From the previous analysis, it could be concluded that the performance of the developed MPA-ANFIS model provided better results than the other models over all the tested datasets. However, the proposed ANFIS-MPA suffered from some limitations, such as its computational time seemed to be higher than other models in some cases. In addition, ANFIS needed some improvement in its structure to avoid the over-fitting problem that occurred when the algorithm was trained using the training set, but it could not provide the optimal response when the testing set was applied to its learned model. Furthermore, the traditional MPA still needed more improvement since it was found that, by analyzing its behavior, the exploitation ability was weaker than the exploration ability.

For more improvement and investigation, the mobility and transportation data between countries and within a country need to be addressed in future work, which may reveal the real reason for this terrifying spread of COVID-19. However, access to these records requires more time.

## 6. Conclusions

With the rapid worldwide spread of SARS-CoV-2 (COVID-19), it is very important to forecast the number of infected people (confirmed cases) to help governments and organizations do the necessary planning to face this severe pandemic. To this end, this study proposed an efficient forecasting model using an enhanced ANFIS model. The MPA was used to optimize the ANFIS parameters. The proposed MPA-ANFIS was used to forecast the number of infected people in four different countries, namely Italy, Iran, Korea, and the USA, using the historical records of these countries that have been updated daily since the beginning of 2020. The evaluation of the proposed MPA-ANFIS was implemented by comparing it to some exiting forecasting models. The outcomes showed that MPA-ANFIS could forecast the number of cases based on the time-series data. Over all the experiments, MPA-ANFIS outperformed all compared models on several measures, such as MAPE, RMSRE, MAE, $R^{2}$, and RMSE.

In future work, the forecasting of the number of confirmed cases of COVID-19 can be improved using the mobility and transportation data of each country, which may explain the rapid rise and spread of the COVID-19. 

## Figures and Tables

**Table 1:** Performance measures.

Measure	Formula
Root Mean Squared Error (RMSE)	$RMSE=\sqrt{\frac{1}{N_{s}}\sum_{i=1}^{N_{s}}{\left(YYP_{i}-Y_{i}\right)}^{2}}$
Mean Absolute Error (MAE)	$MAE=\frac{1}{N_{s}}\sum_{i=1}^{N_{s}}|YYP_{i}-Y_{i}$
Mean Absolute Percentage Error (MAPE)	$MAPE=\frac{1}{N_{s}}\sum_{i=1}^{N_{s}}|\frac{YP_{i}-Y_{i}}{YP_{i}}$
Root Mean Squared Relative Error (RMSRE)	$RMSRE=\sqrt{\frac{1}{N_{s}}\sum_{i=1}^{N_{s}}{\left(\frac{YP_{i}-Y_{i}}{YP_{i}}\right)}^{2}}$
Coefficient of Determination ($R^{2}$)	$R^{2}=1-\frac{\sum_{i=1}^{n}{\left(Y_{i}-YP_{i}\right)}^{2}}{\sum_{i=1}^{n}{\left(Y_{i}-{\bar{Y}}_{i}\right)}^{2}}$

**Table 2:** Parameter settings. FADs, fish aggregating devices. cp, crossover probability. mp mutation probability.

Algorithm	Parameter Setting
ANFIS	Max.epochs=100,errorgoal=0,
	Initialstep=0.01, decreaserate=0.9,
	Increaserate=1.1
GA	Crossovertype=1,
PSO	wMax=0.9,wMin=0.2,C1=2,C2=2
	cp=1, mp=0.01
ABC	a=1,employedbees=N/2,onlookerbees=N/2
SCA	a=2
FPASSA	$Standard\;gamma=1.5,\;Switch\;probability=0.8,C_{2}$∈ [0, 1], $C_{3}$ ∈ [0, 1]
MPA	FADs=0.2,P=0.5,β=1.5

**Table 3:** Results of the USA.

Algorithm	RMSE	MAE	MAPE	RMSRE	*R* ${}^{2}$	Time
ANFIS	80245	58231	744.09	14.0700	0.8371	-
PSO	17656	15545	7.22	0.0801	0.9162	23.24
GA	19302	15846	12.13	0.1624	0.9489	26.23
ABC	345497	335418	1307.52	22.8424	0.7816	44.58
SCA	372321	281297	380.71	5.8893	0.6630	**21.86**
FPASSA	520963	443400	1225.67	18.3782	0.8949	22.98
MPA	**15611**	**12979**	**5.74**	**0.0673**	**0.9595**	45.83

**Table 4:** Results of Iran.

	RMSE	MAE	MAPE	RMSRE	*R* ${}^{2}$	Time
ANFIS	26925.01	21912.08	257.895	5.4871	0.9017	-
PSO	317.99	282.51	0.861	0.0118	0.9861	20.68
GA	**301.39**	271.35	0.840	0.0113	0.9861	23.20
ABC	12581.97	9665.28	51.682	1.0031	0.9111	39.91
SCA	21891.68	14370.36	72.184	1.0982	0.5843	20.24
FPASSA	6830.25	3007.19	28.136	0.6905	0.9457	**20.20**
MPA	302.37	**217.27**	**0.736**	**0.0105**	**0.9874**	39.80

**Table 5:** Results of Italy.

	RMSE	MAE	MAPE	RMSRE	*R* ${}^{2}$	Time
ANFIS	99558.76	81394.93	239.668	5.3878	0.7720	-
PSO	5988.44	4383.08	2.830	0.0368	0.9636	21.59
GA	5772.62	4307.22	2.728	**0.0348**	0.9649	23.75
ABC	99655.58	55053.80	49.518	0.8160	0.7941	40.36
SCA	20644.06	15098.11	138.84	2.6193	0.8622	**20.02**
FPASSA	57704.18	38335.82	101.190	1.7320	0.9278	33.64
MPA	**5465.66**	**3951.94**	**2.634**	0.0372	**0.9859**	39.36

**Table 6:** Results of Korea.

	RMSE	MAE	MAPE	RMSRE	*R* ${}^{2}$	Time
ANFIS	127.76	112.13	1.250	0.0142	0.8228	-
PSO	117.24	88.79	0.979	0.0129	0.8280	20.81
GA	80.01	**60.26**	**0.690**	0.0091	0.9848	24.01
ABC	650.49	399.83	7.886	0.1277	0.7588	35.34
SCA	1145.10	642.06	24.455	0.4701	0.6638	**20.26**
FPASSA	91.68	78.28	0.792	0.0094	0.9038	20.38
MPA	**70.93**	60.31	0.696	**0.0082**	**0.9648**	40.73
